
JOURNAL INFORMATION
==============================
NLM Title Abbreviation: Alzheimers Dement (N Y)
Journal ID: Alzheimers Dement (N Y)
NLM Journal ID: 101650118
Journal ID: TRC2

Alzheimer's & Dementia : Translational Research & Clinical Interventions

EISSN: 2352-8737
Publisher: Wiley

ARTICLE INFORMATION
==============================
PMCID: PMC10865483
DOI: 10.1002/trc2.12395
Article ID: TRC212395
Article version: 1
Subjects: Issue Information, Issue Information

Issue Information

Electronic publication date: 2024 Jan 23
Publication date: 2024 Jan-Mar
Volume: 10
Issue: 1
Electronic Location ID: e12395
Copyright: © 2024 The Authors. Alzheimer's & Dementia: Translational Research & Clinical Interventions published by Wiley Periodicals LLC on behalf of Alzheimer's Association.
License: This is an open access article under the terms of the http://creativecommons.org/licenses/by/4.0/ License, which permits use, distribution and reproduction in any medium, provided the original work is properly cited.
License URL: https://creativecommons.org/licenses/by/4.0/

Figure count: 0
Table count: 0
Page count: 4

source-schema-version-number: 2.0
cover-date: January‐March 2024
details-of-publishers-convertor: Converter:WILEY_ML3GV2_TO_JATSPMC version:6.3.6 mode:remove_FC converted:08.02.2024

==============================

Volume 10, Issue 1, January–March 2024

trci.alzdem.com

### Alzheimer's & Dementia

### Translational Research & Clinical Interventions

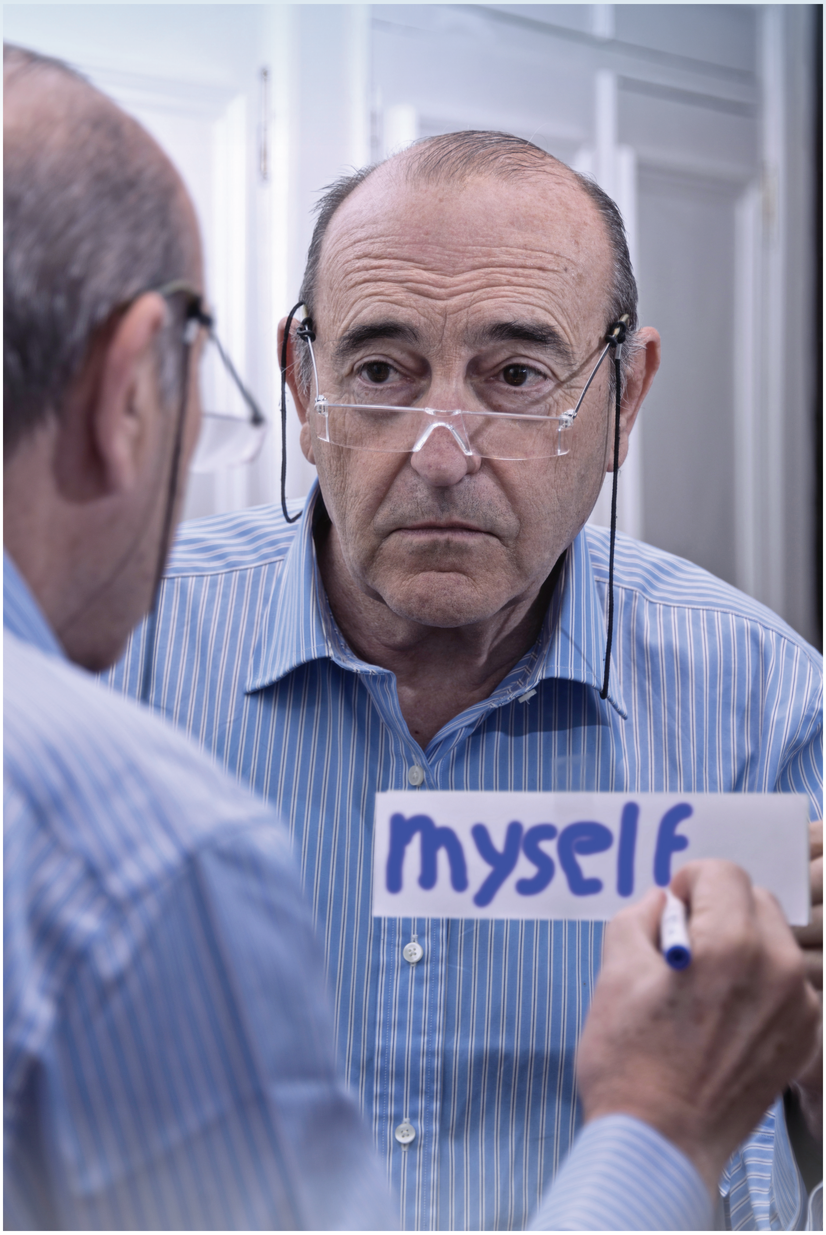

ISSN 2352-8737

WILEY

An open access journal of the

ALZHEIMER'S ASSOCIATION®

Alzheimer's & Dementia

### Translational Research & Clinical Interventions

Volume 10, Issue 1 January-March 2024

### EDITORIAL BOARD

### Editor-in-Chief

Barry D. Greenberg
Baltimore, MD, USA
Email: bgreen45@jhmi.edu

### Deputy Editor

Timothy J Hohman
Nashville, TN, USA
Email: timothy.j.hohman@vumc.org

### Editor-in-Chief Emeritus

Zaven Khachaturian

### Executive Editor Emeritus

Ara Khachaturian

### Founding Editor-in-Chief

Lon Schneider

### Consulting Editors

Donna M. Wilcock, Indianapolis, IN, USA
Liana Apostolova, Indianapolis, IN, USA
Peter J. Snyder, Kingston, RI, USA

### Senior Associate Editors

Lisa L. Barnes, Chicago, IL, USA
Luc Buée, Lille, France
Peggy Dilworth-Anderson, Chapel Hill, NC, USA
Hiroko Dodge, Boston, MA, USA
Malú Gámez Tansey, Gainesville, FL, USA
Sam Gandy, New York, NY, USA
Andrea Gilmore Bykovskyi, Madison, WI, USA
Walter Kukull, Seattle, WA, USA
Cynthia A. Lemere, Boston, MA, USA
Michelle Mielke, Winston-Salem, NC, USA
Niels Prins, Amsterdam, the Netherlands
Marwan Noel Sabbagh, Phoenix, AZ, USA
Mary Sano, Bronx, NY, USA
Dominic Trepel, Dublin, Ireland

Alzheimer's & Dementia

### Translational Research & Clinical Interventions

Volume 10, Issue 1

January-March 2024

### Associate Editors

Steve Arnold, Cambridge, MA, USA
Laura Baker, Winston-Salem, NC, USA
Sarah Banks, San Diego, CA, USA
Jessica Caldwel, Las Vegas, NV, USA
Ryan Dougherty, Baltimore, MD, USA
Maria Glymour, San Francisco, CA, USA
Michael Gold, Chicago, IL, USA
Benjamin Hampsted, Ann Arbor, MI, USA
Dirk Keene, Seattle, WA, USA
Michael Lutz, Durham, NC, USA
Arjun Masurkur, New York, NY, USA
Sid O'Bryant, Fort Worth, TX, USA
Adesole Ogunniyi, Ibadan, Nigeria
Esther Oh, Baltimore, MD, USA
Van Park, San Francisco, CA, USA
Rema Raman, San Diego, CA, USA
Stacy Rizzo, Pittsburg, PA, USA
Keenan Walker, Baltimore, MD, USA
Henrik Zetterberg, Stockholm, Sweden

### Editorial Board

Jose Abisambra, Gainesville, FL, USA
Suvarna Alladi, Bangalore, India
Wyllians Vendramini Borelli, Porto Alegre, Rio Grande do Sul, Brazil
Greg Carter, Bar Harbor, ME, USA
Susan Catalano, South San Francisco, CA, USA
Ding Ding, Shanghai City, China
Claudia Duran-Aniotz, Santiago, Chile
Lutz Frölich, Heidelberg, Germany
Elizabeth Head, Irvine, CA, USA
Karl Herrup, Pittsburgh, PA, USA
Gareth Howell, Bar Harbor, ME, USA
Jianping Jia, Beijing, China
Catherine Kaczorowski, Ann Arbor, MI, USA
Raj Kalaria, Newcastle upon Tyne, United Kingdom
Thomas Karikari, Pittsburgh, PA, USA
Melissa Lamar, Chicago, IL, USA
Frank Longo, Palo Alto, CA, USA
Mychael Lourenco, Rio de Janeiro, Brazil
Colin Masters, Parkville, Victoria, Australia
Abhay Moghekar, Baltimore, MD, USA
Graciela Muniz-Terrera, Edinburgh, Scotland
Adrian Oblak, Indianapolis, IN, USA
Chiadi Onyike, Baltimore, MD, USA
Chengxuan Qiu, Stockholm, Sweden
Paul Rosenberg, Baltimore, MD, USA
Quincy Samus, Baltimore, MD, USA
C. Elizabeth Shaaban, Pittsburgh, PA, USA
Yong Shen, Beijing, China
Carmela Tartaglia, Toronto, Canada
Paul Territo, Indianapolis, IN, USA
Ronald Thomas, La Jolla, CA, USA
Li-San Wang, Philadelphia, PA, USA
Eduardo Zimmer, Porto Alegre, Brazil

Alzheimer's & Dementia

### Translational Research & Clinical Interventions

**Open Access and Copyright**

All articles published by *Alzheimer's & Dementia: Translational Research & Clinical Interventions* are fully open access: immediately freely available to read, download, and share. All articles are published under the terms of a Creative Commons license.

Copyright on any research article published by *Alzheimer's & Dementia: Translational Research & Clinical Interventions* is retained by the author(s).

Further information about open access licenses and copyright can be found at: https://authorservices.wiley.com/author-resources/Journal-Authors/licensing/licensing-info-faqs.html#4. Information on the licenses used by *Alzheimer's & Dementia: Translational Research & Clinical Interventions* can be found at https://alz-journals.onlinelibrary.wiley.com/.

**Disclaimer**

The Publisher, Alzheimer's Association, and Editors cannot be held responsible for any errors or any consequences arising from the use of information contained in this journal; the views and opinions expressed do not necessarily reflect those of the Publisher, Alzheimer's Association, or Editors, neither does the publication of advertisements constitute any endorsement by the Publisher, Alzheimer's Association, Editors, or Authors of the products advertised. Wiley Open Access articles posted to repositories or websites are without warranty from Wiley of any kind, either express or implied, including, but not limited to, warranties of merchantability, fitness for a particular purpose, or non-infringement. To the fullest extent permitted by law Wiley disclaims all liability for any loss or damage arising out of, or in connection with, the use of or inability to use the content.

For submission instructions and all other information visit: https://alz-journals.onlinelibrary.wiley.com/

Cover Image Credit: Shutterstock / Fresnel
